# Bulat-Klarica-Oreskovic Hypothesis: A Comprehensive Review

**DOI:** 10.7759/cureus.45821

**Published:** 2023-09-23

**Authors:** Joe Bajda, Neharaj Pitla, Vasavi Rakesh Gorantla

**Affiliations:** 1 Neurology, St. George’s University, St. George's, GRD; 2 Biomedical Sciences, West Virginia University School of Osteopathic Medicine, Lewisburg , USA

**Keywords:** lymphatics of the brain, csf dynamics, hydrocephalus, glymphatics, intracranial hypertension, cerebrospinal fluid, cerebrospinal fluid pathophysiology

## Abstract

Classical theories of cerebrospinal fluid (CSF) production and flow are taught throughout medical education. The idea that CSF is produced and/or filtered by the choroid plexus and flows in one direction throughout the ventricular system has been a largely accepted thesis. However, modern studies have called into question the validity of this hypothesis, suggesting that CSF does not move unidirectionally but rather is driven by microvessel contractions in a to-and-fro manner throughout the cerebrospinal system. Moreover, new insights suggest that in addition to CSF production, the exchange of fluids and proteins between the cortical vasculature and the interstitium may function as the brain’s version of a lymphatic system. This comprehensive review provides evidence for a different framework of CSF flow. One that includes perivascular pulsations that push CSF back and forth, allowing exchange between the CSF and interstitium, and with CSF production occurring throughout the cerebrospinal system. These findings could be revolutionary in understanding the pathophysiology of CSF flow and in the treatment of pathologies such as intracranial hypertension, hydrocephalus, Alzheimer's disease, and many others.

## Introduction and background

Cerebrospinal fluid (CSF) is a clear, colorless fluid usually rich in glucose (60%-80%) [1]. Classical studies suggest that most of the CSF is secreted and/or filtered by the choroid plexus within the brain’s ventricular system [2]. Proposed in 1914 by Lewis Weed, Walter Dandy, and Harvey Cushing in the “Classic” or Weed-Dandy-Cushing Hypothesis [3], CSF is believed to be circulated from the ventricles to the subarachnoid space and is eventually distributed around the spinal cord [1]. This hypothesis advocates for the unidirectional flow of CSF [2]. CSF pathologies, such as hydrocephalus, have been treated using this framework, and it is still taught in medical education today. The unidirectional flow was thought to explain why if one pathway, such as one of the interventricular foramen, is obstructed the contralateral lateral ventricle will expand. However, recent experimental data contradict this previous theory [3]. 

Bulat et al. described the multidirectional flow of CSF based on fluid mechanics in the central nervous system (CNS) microvessels [4]. This hypothesis suggests that pulsations from the cerebral microvessels propel the exchange of fluid between CSF and the interstitium. Moreover, it asserts that CSF is not produced in any significant amount by the choroid plexus but rather can be produced anywhere throughout the cerebrospinal system [4]. Components of CSF can even reach the systemic circulation, suggesting a full-body equilibrating mechanism. This model is called the Bulat-Klarica-Oreskovic hypothesis and represents the modern explanation for CSF physiology. The exchange between the interstitium and CSF allows for the clearance of cerebral waste proteins and serves as the brain’s equivalent of a lymphatic system. This construction is a significant deviation from the classical model proposed by Weed-Dandy-Cushing [3-8]. 

Understanding the process of CSF physiology is essential for the management of CNS disorders. The Bulat-Klarica-Oreskovic model opens the possibility for new approaches to diseases that involve CSF. Hydrocephalus is an illustrative example of the discrepancies between each model. It is defined as an accumulation of CSF that causes the ventricles to swell. It can be communicating, meaning without obstruction, or obstructive, with obstruction. Hydrocephalus is treated based on the classical model, but experiments that have led to the development of the modern hypothesis demonstrate that the success of some procedures may be merely coincidental [9,10]. Suppose that there is a buildup of CSF causing expansion of the ventricles, the classical theory suggests that there is only one way for the CSF to drain, throughout the ventricular system and ultimately into the subarachnoid granulations. Therefore, one of the logical responses to this is to create a shunt, called a ventriculoperitoneal (VP) shunt, to make an alternate pathway for drainage. Javeed et al. showed that in 86.5% of cases (n=1030) there was no postoperative hydrocephalus following VP shunt. This study concluded that clinically good outcomes occurred in 63.4% of cases, which they describe as avoiding complications and mortality [11]. Despite a high rate of complications, it seems that this is at least a moderately successful procedure, so how would the modern hypothesis explain this success? Modern thought might suggest that acute changes in CSF volume or pressure would also affect the surrounding capillaries. One theory is that changes in impedance cause vascular pulsations to become more rapid and relocate more proximally, causing an inversion of CSF flow leading to ventricular enlargement that is characteristic of hydrocephalus. These alterations would not necessarily contradict the findings of classical theories of CSF flow but rather establish an additional dependence upon cardiac pulsations and rigidity of the cerebral vasculature [12]. There are many other modern proposals for the mechanisms of CSF pathophysiology, including CSF flow and clearance of proteins that would be important for understanding numerous pathologies. These were withheld from this review as we have tried to focus on the chronological discoveries pertaining to the flow and production of CSF rather than an exhaustive analysis of the potential consequences of these findings. Nonetheless, it is important to further our understanding of the pathophysiology of the CNS so that we can treat the underlying mechanism of disease.

As always, new ideas bring exciting possibilities and the hope that challenging pathologies can finally be conquered, but it is important to evaluate these new theories with a critical mind. This review aims to summarize the proposed mechanisms of CSF dynamics to support the findings by Bulat et al. [4,9].

## Review

Results

While the classical model has been largely accepted since its publication in the early 1900s, there have been several notable studies in the subsequent years that have pushed incrementally toward the modern hypothesis. In this comprehensive review, we have included studies supporting the modern hypothesis based on relevance and estimated influence on the progression toward current theory. Studies were included based on searches for modern CSF pathophysiology, modern CSF theory, CSF dynamics, glymphatics, and the brain’s lymphatic system. Studies that were not available in full text or English were excluded. 

Some of the first deviations from classical theory were seen in studies performed by Bering et al., Chiro et al., Naidich et al., and Drayer et al. The significance was unknown at the time, but these studies suggested that CSF may not be exclusively, or even primarily, produced within the choroid plexus. Moreover, they opened the possibility that CSF exchange may occur between the perivascular networks within the brain and systemic circulation [13-17]. Conner et al. continued the exploration of CSF physiology by studying normal pressure hydrocephalus in cats. Pressure gradients between cerebral structures were found despite stable ventricular pressures, suggesting additional mechanisms for establishing equilibrium of CSF flow [18]. Shapiro et al. expanded on the exploration of CSF physiology by studying obstructive hydrocephalus in cats. Unique to this experiment was that, as pressure built up within the ventricular system, the pressure gradient between the ventricles and the subarachnoid space (transmantle gradient) dissipated. Again, this seemed to indicate an equilibrating mechanism beyond the choroid plexus [19]. In 1991, Castro et al. studied transcortical circulation and its relationship with the surrounding interstitium. It was discovered that during hydrocephalus, pressures in the ventricular system were transmitted to the surrounding vasculature. Synchronous pressure changes were thought to show an interdependence between the ventricular system and the cortical circulation [20]. 

The first published description of the modern hypothesis occurred in 1993 when Bulat et al. proposed that CSF flow is not limited to unidirectional flow [4]. In 2008, Bulat and colleagues performed an experiment to demonstrate this hypothesis. This experiment, reinforced a couple of years later in a study by Oreskovic and Klarica, illustrated an exchange of CSF between the ventricular system and the perivascular space [9,21]. Igarashi et al. moved a step further in the understanding of CSF physiology by demonstrating the dependence of CSF flow on aquaporin-4 channels [22,23]. Several studies have since used more advanced imaging techniques, such as time-slip MRI, to better visualize CSF flow. These studies created higher resolution images that demonstrated rapid exchange of CSF throughout cortical spaces, again suggesting that the ventricular system must be in contact with the cerebrum [7,24-26]. These findings are summarized in Table 1.

**Table 1:** Chronology of the published papers that challenged the “Classic” or Weed-Dandy-Cushing Hypothesis

Year	Author	Findings	Conclusions
1952	Bering et al. [13]	Demonstrated that large amounts of CSF uptake occur in the periventricular white matter. Di Chiro suggested at this time that CSF production can occur anywhere throughout the CSF circulation, denying that it’s only produced in the choroid plexus.	CSF may not exclusively, or even predominantly, be produced by the choroid plexus. Uptake of CSF into the periventricular white matter allows for the possibility of systemic exchange through the perivascular system.
1964	Chiro et al. [14]		
1976	Chiro et al. [15]		
1976	Naidich et al. [16]		
1977	Drayer et al. [17]		
1984	Conner et al. [18]	Demonstrated an increase in transmantle pressure during normal pressure hydrocephalus.	Pressure gradients may be distributed throughout the cerebral structures despite stable pressure within the ventricular system.
1987	Shapiro et al. [19]	Ventricular expansion can occur in obstructive hydrocephalus without a measurable transmantle gradient.	Suggests an equilibrating mechanism to dissipate pressure.
1991	Castro et al. [20]	Elevated cortical vein pressure contributes to the formation and/or maintenance of hydrocephalus. CSF exchange includes transcapillary and transvenous absorption from the interstitial space.	Perivascular pressure elevation during hydrocephalus is indicative of the relationship between ventricular fluid and cortical blood flow. Synchronous pressure elevation suggests a dependent interrelation.
1993	Bulat et al. [4]	Proposes a new hypothesis for CSF exchange.	This was the first published description of the modern hypothesis. It proposed the idea that CSF did not strictly travel unidirectionally.
2008	Bulat et al. [9]	Demonstrated that CSF does not flow unidirectionally, but rather flows bidirectionally within the perivascular space. Flow is driven by pulsations of the cerebral vasculature.	CSF exchange via vascular pulsations illustrates a mechanism similar to other areas of the body. If true, pathologies such as obstructive hydrocephalus would need to be reviewed.
2010	Oreskovic and Klarica [21]		
2010	Bulat and Klarica [27]		
2013	Igarashi et al. [22]	Igarashi and colleagues demonstrate in knockout mice that water flow into the cerebrospinal fluid is heavily dependent upon aquaporin-4 channels.	Permeability of aquaporin-4 channels, and not the choroid plexus, are the major drivers of CSF exchange.
2014	Igarashi et al. [23]		
2017	Takeuchi et al. [24]	Use of Time-Slip MRI to more rapid visualization of the movement of CSF throughout the cortical spaces and the body.	Higher resolution imaging allows visualization of CSF throughout the cortical spaces, disputing the notion that CSF is traveling unidirectionally throughout the ventricular system. Fluids traveling along the deep cortical spaces with the inner cerebral vasculature must be in communication with the CSF held within the ventricles.
2018	Shibukawa et al. [25]		
2021	Hablitz et al. [7]	Used advanced imaging techniques to visualize bi-directional CSF flow.	CSF synthesis and secretion is not limited to the choroid plexus, but rather can be produced at multiple cortical sites. Moreover, CSF flow is multidirectional, coinciding with the modern hypothesis years earlier.
	Yamada et al. [26]		

Expanded consideration of results

In 1952, Bering published an article demonstrating that large amounts of CSF uptake occur in the periventricular white matter. Deuterium oxide (D2O, heavy water) with 0.8% NaCl was injected into animals (monkeys and dogs) and humans and subsequently monitored. It was discovered that the fluid rapidly accumulated in the cisterna magna and lumbar subarachnoid space before equilibrating within the ventricles and the remainder of the lumbar areas. This suggested that CSF was not produced solely by the choroid plexus, otherwise, the cerebral ventricles would be the first areas to accumulate the D2O tracers. Moreover, patients who underwent choroid plexectomy were found to have negligible alteration in water exchange [13]. 

Considerable controversy remained about the pathway of CSF exchange. In 1964, Chiro et al. referenced radiolabeled iodine tracer studies demonstrating the possibility of CSF production throughout the cerebrospinal system. Although Chiro admitted at this time, the net of flow is likely forward through the traditional ventricular progression, he added that at least part of the fluid must move in a to-and-fro fashion, with vascular pulsations as a likely mechanism [14]. 

Naidich et al., in 1976, explored periventricular edema in the setting of hydrocephalus using computerized tomography (CT) imaging. The study suggested that hydrocephalus primarily affects the white matter, while sparing the gray matter. Images from this study revealed minimal disruption of the caudate nucleus and thalamus but significant disruption of the white matter, including fragmentation and swelling. Degeneration of the cortical mantle and edematous changes caused by hydrocephalus led to neuron loss [16]. In 1977, Drayer et al. performed an analysis of the kinetics of CSF flow, once again using CT imaging. Metrizamide was given intrathecally in patients with communicating hydrocephalus, who were then monitored via CT cisternography both for the flow of CSF, and to determine the cause of hydrocephalus. It was shown that metrizamide could be a safe, and perhaps better, alternative to radionuclide dyes previously used for visualization of cerebral CSF flow. Although the goal of Drayer and colleagues was to demonstrate the utility of CT imaging for diagnosis and pathophysiology understanding, much like Naidich et al., they also revealed evidence that significant volumes of CSF uptake occur within the periventricular white matter [17]. 

Conner et al., in 1984, published research on normal pressure hydrocephalus and its relationship to transmantle pressure. Hydrocephalus was induced in cats using intracisternal injection of kaolin, allowing for measurements comparing the ventricular pressure to the transmantle pressure. Pressure over the convexity of the cerebrum (transmantle) was shown to be elevated while ventricular pressure remained stable over a four-week period, suggesting that transmantle pressure was a determinant of ventricular dilation. Moreover, it was hypothesized that this could be an explanation for pseudotumors, in which the ventricles do not dilate despite increasing pressure [18]. Cats with intracisternal injection of kaolin were used by Shapiro et al. in 1987 to research transmantle pressure in hydrocephalus [19]. Interestingly, this study found that there was no discernable gradient between transmantle and ventricular pressure, concluding that ventricular expansion can increase despite stable pressure over the cerebral convexity. The authors address the contradiction with Conner et al., suggesting that because their study extended the time of observation to nine weeks, it is indicative of the pressure gradients attenuating over time. The scientific community at this time largely adhered to the classical thought process of unidirectional flow and choroidal CSF production, but a noteworthy footnote from this study is that whether fluid was injected into the cisterna magna or the ventricles, all the intracranial pressures (ICPs) changed in unison. Likewise, the pressures equilibrated to a steady state together. Questions about the mechanisms for ICP compensation remained, but this study demonstrated that a change in neuroaxis volume induces equilibration of pressures within the intracranial spaces [19]. A few years later, in 1991, Castro et al. sought to further understand CSF hydrodynamics and their relationship with the cerebrovascular system by examining cortical pressures within naturally hydrocephalic dogs. Unique to this study was the addition of measuring cortical vein pressure, while also comparing transmantle and ventricular pressures. It was demonstrated that in hydrocephalic dogs the gradient between transmantle pressure and the sagittal sinus was not greatly altered, but cortical vein pressure was markedly elevated. This suggested that cortical vein pressure plays a role in the development and maintenance of hydrocephalus, implicating transcapillary and transvenular exchange between CSF and the interstitium and thus paving the way for the beginnings of the modern hypothesis [20].

Bulat et al. officially proposed the modern idea for CSF flow in 1993, in a conference paper entitled, “Dynamics and Statics of the Cerebrospinal Fluid: The Classical and a New Hypothesis”. The author of many papers and extensive research in cerebral biochemistry and pathophysiology, Bulat hypothesized what many of the aforementioned experiments suggested; CSF does not flow unidirectionally, but in fact travels via pericapillary mechanisms in a to-and-fro manner [4]. This was a significant deviation from the traditional explanation and it would take years of experimentation, including from Bulat himself, to gather supportive empirical data.

In 2008, Bulat and his colleagues Lupret, Oreskovic, and Klarica clarified this by hypothesizing that exchange of macromolecules occurs in a bidirectional fashion between the interstitium and perivascular spaces. Moreover, they suggested that extracellular fluid, mainly water, is transferred by cerebral microvessels with systolic-diastolic pulsations. Pathways of macromolecules and water were compared using 3H-Inulin and 3H-water, respectively. 3H-water infusions into the ventricles coincided with significant pressure increases in the cerebral arterial plasma and cisternal CSF, suggestive that 3H-water is absorbed from the ventricles into the periventricular capillaries, which ultimately drain into the confluence of sinuses. Concordantly, the amount of 3H-water that had drained into the venous circulation was greater than what remained in the cisterns and arterial plasma. This provided a roadmap for 3H-water flow from the confluence of sinuses to the systemic circulation, before returning across the cerebral capillary walls into the CSF and interstitial fluid, establishing rapid equilibrium. 3H-Inulin was injected into the lateral ventricles, while once again measuring its presence within the arterial plasma, cisterns, and confluence. In this case, concentrations within the cisterns increased, but 3H-Inulin in the arterial plasma and confluence remained near the baseline. 3H-Inulin was demonstrated to be slowly removed from the CSF into the systemic circulation before ultimately being excreted in the urine [9]. Following this experiment, two reviews were written in 2010 challenging the old hypothesis and providing new insights into the hydrodynamics of CSF. One by Bulat and Klarica, the other by Oreskovic and Klarica. These papers described the contradictions the Dandy-Weed-Cushing model now faces through experimentation. They asserted that CSF travels multidimensionally using the larger surface area along the pericapillary space, which explained the rapid equilibration between the arterial plasma and the confluence of sinuses. Pulsations from the periventricular capillaries drive fluid in a to-and-fro fashion, causing exchange of CSF and interstitial fluid before ultimately draining into the venous sinuses. The underlying physiological conditions within each CSF compartment, including changes in ICP, influence this exchange of fluid. Moreover, referring to previous experiments, they asserted that the majority of CSF is not produced by the choroid plexus, but is produced and absorbed throughout the whole CSF system [21,27].

The next logical step seemed to be figuring out the mechanism by which fluid is exchanged between the perivascular spaces and interstitium. Igarashi et al. performed a pair of studies using aquaporin-4 knockout mice, demonstrating the dependence of fluid exchange upon these channels. An aquaporin-4 inhibitor, TGN-020 (2-nicotinamido-1,3,4-thiadiazole) was administered to control mice and knockout mice to compare the effects on regional cerebral blood flow. Following administration of TGN-020, the knockout mice exhibited very little change in regional cerebral blood flow, while the wild type showed a significant increase. Acetazolamide administration, used as a positive control, resulted in an even greater increase in regional cerebral blood flow in the knockout mice, with a similar increase in the wild-type group. Igarashi et al. then performed a similar study, this time including aquaporin-1 and aquaporin-4 knockout mice to measure radiolabeled water influx in the CSF, cortex, and basal ganglia. It was shown that aquaporin-1 inhibition caused minimal change in water influx into the CSF, suggesting that these channels play little role in this process. Aquaporin-1 channels are nearly completely restricted to the choroid plexus, indicating that regulation of water exchange within the CSF is not driven by these channels, but rather more dependent upon aquaporin-4 channels located diffusely throughout the astrocytic endfeet along transcerebral capillaries. CSF carriage of solutes from the brain parenchyma along the perivascular space seemed to be the brain’s equivalent to the lymphatics system and may provide a route for clearance of pathological macromolecules, such as amyloid proteins found in Alzheimer’s disease [22,23]. This framework illustrates CNS lymph drainage as functioning much like the rest of the body with solutes passing through the perivascular space before being drained into the deep cervical lymph nodes, and then into right lymphatic and thoracic ducts [26,28]. 

Modern explorations into the hydrodynamics of the brain include time-slip MRI imaging to more rapidly view CSF changes within the cerebral spaces. Studies by Yamada et al., Takeuchi et al., Shibukawa et al., and several others have used this technology to better understand cerebral pathophysiology. One of the benefits of this technology is that it allows CSF itself to be used as the tracer, rather than relying on radiolabeled analogues, thus preserving natural physiology. Notable from these studies is that CSF is not shown to travel unidirectionally, but rather pulsates back and forth, moving cranially during diastole and caudally during systole, as the Bulat-Klarica-Oreskovic theory hypothesized years earlier. Visualization of these pulsations has been made using time-slip MRI, with a seemingly predictable relationship with stroke volume emerging. Moreover, acceleration of waste removal has been demonstrated during shaking of the head and sleep, suggesting physiological conditions play a role in CSF exchange [24-26]. Further research is needed to explain why and if this is the case, considering these are two seemingly opposite physiological states. Yamada et al. suggested this may be a selective process, reporting that only the CSF adjacent to the circumventricular organs shows increased movement at rest compared to the remainder of the cerebrospinal system [26]. Nonetheless, each of these studies indicates an evolving understanding of CSF pathophysiology beyond the choroid plexus-dominated model of classical theory.

Limitations

Although we have tried to be inclusive of the major chronological discoveries relating to modern CSF pathophysiology, the studies included are not exhaustive. There are studies that may provide evidence supporting and contradicting the modern hypothesis that was not found in the selection process. Moreover, the importance of discoveries was determined based on relevance to the topic and estimated influence on the progression toward the modern theory. These metrics are subjective and limit the use of this study to a summary of discoveries rather than an analysis of the findings of each study. 

## Conclusions

The study of ICP and cerebrospinal fluid dynamics has progressed significantly in the past century. Evidence that CSF flow is multidirectional and that production occurs throughout the cerebrospinal system is accumulating. Techniques like CT imaging, aquaporin-4 knockout mice, radiolabeled tracers, and advanced MRI imaging have provided evidence supporting multidirectional CSF exchange. Modern research points toward vascular pulsations driving the exchange of fluid between the interstitium and CSF, thus providing a new framework for CSF dynamics. If the modern models are accurate, one could imagine the implications in treating conditions such as hydrocephalus, intracranial hypertension, and neurodegenerative diseases like Alzheimer's. Although the reviewed studies provide convincing evidence, the understanding of CSF dynamics is still evolving, and additional research is needed to elucidate the mechanisms involved more completely. It would be helpful to have contemporary studies examining the equilibration mechanisms suggested by earlier research. We now have access to technology that could provide a more instantaneous view and perhaps unlock ways to manipulate CSF exchange when desired. Moreover, research into the clearance of CSF proteins has begun, but experimentation designed to understand the drivers of waste clearance would provide hope toward treating pathologies hallmarked by the accumulation of protein (Alzheimer's disease, Parkinson's disease, etc.).
